# Timed “up and go” to identify physically inactive individuals with interstitial lung disease

**DOI:** 10.36416/1806-3756/e20240248

**Published:** 2025-03-18

**Authors:** Camile Ludovico Zamboti, Larissa Dragonetti Bertin, Gabriela Garcia Krinski, Humberto Silva, Heloise Angélico Pimpão, Emanuel Gois, Fabio Pitta, Carlos Augusto Camillo

**Affiliations:** 1. Departamento de Fisioterapia, Laboratório de Pesquisa em Fisioterapia Respiratória - LFIP - Universidade Estadual de Londrina, Londrina (PR) Brasil.; 2. Departamento de Fisioterapia, Faculdade de Ciências e Tecnologia, Universidade Estadual Paulista - UNESP - Presidente Prudente (SP) Brasil.; 3. Departamento de Ciências da Reabilitação, Universidade Pitágoras - UNOPAR - Londrina (PR) Brasil.; 4. Departamento de Clínica Cirúrgica da Universidade Estadual de Londrina, Londrina (PR) Brasil.

**Keywords:** Physical functional performance, Lung diseases, interstitial, Activities of daily living

## Abstract

**Objective:**

To investigate the relationship between the timed “up and go” (TUG) test and physical activity in daily life (PADL) in patients with interstitial lung disease (ILD) and propose a cutoff point to identify physically inactive individuals.

**Methods:**

Participants performed the TUG test at a usual pace (TUG_usual_) and at a fast pace (TUG_fast_). Exercise capacity was assessed by the six-minute walk test, lung function was assessed by whole-body plethysmography, quadriceps strength was assessed by maximal voluntary isometric contraction, and PADL was assessed by an activity monitor worn for six consecutive days. PADL variables included number of steps/day, time spent/day in activities of different intensities, and time spent/day in different postures. A ROC curve was plotted to identify physically inactive individuals on the basis of daily steps (5,000 steps/day) and moderate to vigorous physical activity (MVPA; 30 min/day).

**Results:**

Fifty-three ILD patients (26 women, with a mean age of 60 ± 11 years) were included in the study. TUG_usual_ and TUG_fast_ correlated moderately with the number of steps/day and time spent/day in light physical activity and MVPA (−0.60 < r < −0.41; p < 0.05 for all). ROC curves for TUG_usual_ showed that the cutoffs of ≥ 9.25 s and ≥ 7.9 s can identify physically inactive individuals on the basis of 5,000 steps/day (AUC: 0.73; sensitivity, 76%; specificity, 70%) and 30 min/day of MVPA (AUC: 0.85; sensitivity, 90%; specificity, 75%). Participants who performed worse on TUG_usual_ (i.e., ≥ 9.25 s) showed lower peripheral muscle strength, exercise capacity, and PADL.

**Conclusions:**

Performance on TUG_usual_ and TUG_fast_ correlates moderately with PADL in patients with ILD. A TUG_usual_ performance ≥ 9.25 s appears to be able to identify physically inactive individuals in this population.

## INTRODUCTION

Patients with interstitial lung disease (ILD) can experience progressive loss of lung function and physical performance, as well as worsening of symptoms and deterioration in health-related quality of life.^1^ It is increasingly recognized that extrapulmonary manifestations are associated with worse prognosis in patients with ILD.^2^ Extrapulmonary manifestations include low levels of physical activity in daily life (PADL), which are known to be present in patients with respiratory conditions.^3^ Inactivity plays a critical role in the vicious cycle of chronic respiratory diseases and is associated with worse prognosis in patients with idiopathic pulmonary fibrosis.^4^

Reduced exercise capacity and muscle strength are common in patients with ILD^5^ and contribute to reducing their ability to perform daily functional tasks.^6^ Therefore, in addition to PADL, functional performance is increasingly assessed in patients with chronic respiratory diseases,^7^ mainly by functional tests such as the timed “up and go” (TUG) test. The TUG test is reliable in patients with ILD, assessing exercise capacity and muscle strength^8^ through a series of tasks necessary for independent living, such as walking, sitting/standing, and changing directions^9^ at a usual pace (TUG_usual_) or as fast as possible (TUG_fast_). In patients with chronic respiratory diseases other than ILD, the TUG test is used in order to assess functional mobility, walking ability, and dynamic balance,^10^ as well as clinical outcomes such as the risk of falling.^11^ Additionally, performance on the TUG test reflects disease severity and is responsive to pulmonary rehabilitation in patients with COPD.^12^ ^,^ ^13^

Functional performance tests are generally simple and practical. Because accelerometers are not readily available in clinical practice, instruments that can accurately assess PADL in the clinical setting are important.^14^ ^,^ ^15^ Functional performance and PADL have been reported in association with respiratory conditions.^16^ ^-^ ^18^ However, there are currently no studies exploring the relationship between the TUG test and PADL in ILD patients. Therefore, a cutoff point to identify inactivity in ILD patients could guide clinicians in their decision to investigate PADL or intervene when necessary. 

Given that the TUG test requires the ability to walk and perform movements that are commonly performed during activities of daily living, and given that patients with ILD show significantly lower levels of PADL than do those with other respiratory diseases,^19^ ^,^ ^20^ we hypothesized that the TUG test might be associated with PADL and sedentary behavior in patients with ILD. Furthermore, we hypothesized that the performance of ILD patients on the TUG test could be used in order to identify physically inactive individuals. Therefore, the objective of the present study was to investigate the relationship between the TUG test and PADL in patients with ILD and propose a cutoff point to identify physically inactive individuals. 

## METHODS

This cross-sectional study was part of a larger trial conducted in the outpatient clinic of the University Hospital of the State University at Londrina, located in the city of Londrina, Brazil. The study was approved by the local institutional review board (CAAE no. 69598317.5.0000.5231), and all participants gave written informed consent. 

The convenience sample included patients who had been diagnosed with ILD in accordance with international guidelines^21^ and who had been clinically stable (i.e., with no respiratory exacerbations) for at least one month before recruitment. The inclusion criteria were being in the 40- to 75-year age bracket and being clinically able to undergo testing. Patients who at the time of testing presented with comorbidities that could affect their functional performance-comorbidities such as cognitive deficit and pain-were excluded, as were those who presented with respiratory diseases (as assessed by pulmonary function tests) and those who withdrew consent. 

All participants answered a sociodemographic questionnaire. Participants then underwent the TUG test twice at a usual pace (TUG_usual_) and twice at a fast pace (TUG_fast_).^22^ During the tests, individuals were requested to stand up from a chair; walk a distance of 3 m at a usual pace (TUG_usual_) or as fast as possible (TUG_fast_); and then turn and walk back to the chair at the same pace to sit down again.^23^ TUG_usual_ reflects the majority of tasks performed in daily life and is the more commonly used test,^12^ whereas TUG_fast_ reflects the greatest speed at which an individual can perform activities of daily living, being more closely associated with the risk of falls.^24^

Participants were allowed to use walking aids and oxygen.^25^ For those who required oxygen therapy, a trained physical therapist was recruited to carry the oxygen delivery device.^25^ ^,^ ^26^ The time in seconds to complete the test was recorded and used as the primary outcome measure. A stopwatch was used in order to time the tests. The stopwatch was started when participants got up from the chair and stopped when they sat down again after the three-meter walk.^8^ Faster walking speeds indicated better mobility. The faster of two attempts was used for analysis of the TUG_usual_ and TUG_fast_ tests, in accordance with previous validation studies reporting a significant learning effect between tests.^8^ ^,^ ^25^ ^,^ ^26^ Participants were allowed to rest between tests until their heart rate and SpO_2_ returned to baseline values or until they confirmed that they were ready to proceed.^25^ Reference equations for the Brazilian population were used in order to analyze performance on the TUG_usual_ and TUG_fast_ tests, expressed as a percentage of the predicted value.^27^

The levels of PADL were assessed with an activity monitor (wGT3X-BT^®^; ActiGraph LLC, Pensacola, FL, USA), which participants wore on their waist 24 h a day for six consecutive days. The aforementioned activity monitor has been validated for use in patients with respiratory diseases, being a reliable method to assess PADL.^28^ The assessment was considered valid if participants wore the monitor for at least 8 h/day for four weekdays.^29^ Participants were instructed to remove the monitor during water activities. The device measures wearing time and records daily steps, time spent in different postures (i.e., lying, sitting, and standing), and time spent in physical activity of different intensities during waking hours, as follows: sedentary behavior, < 1.5 metabolic equivalents of task (METs); light physical activity (LPA), between 1.5 and 3 METs; and moderate to vigorous physical activity (MVPA), > 3 METs.^29^ Data on PADL were analyzed with ActiLife^®^ software (ActiGraph LLC). Inactivity was defined as < 30 min/day of MVPA^30^ and < 5,000 steps/day.^31^ ^,^ ^32^

Exercise capacity was evaluated by the six-minute walk test (6MWT), which was performed in accordance with international guidelines,^33^ with a 30-min rest between tests. The longest six-minute walk distance (6MWD) was used for analysis, being compared with normative values. One week later, participants returned to the laboratory to return the activity monitor and undergo lung function and peripheral muscle strength assessment. Lung function was assessed by post-bronchodilator spirometry, whole-body plethysmography, and DL_CO_ measurement, all of which were performed with a Vmax plethysmograph (CareFusion, San Diego, CA, USA) and in accordance with international guidelines.^34^ ^-^ ^37^ The obtained values were compared with normative data for the Brazilian population.^38^ Quadriceps strength was assessed by maximal voluntary isometric contraction of the dominant limb, with the use of a strain gauge (EMG System do Brasil, São José dos Campos, Brazil) attached to a multigym. Participants were instructed to perform a maximal voluntary isometric contraction for 6 s, with 90° hip and knee flexion. At least four and at most 15 attempts were made, and the highest value was used for analysis. Finally, handgrip strength of the dominant hand was evaluated with a handheld dynamometer (SH1001; Saehan Corporation, Changwon, South Korea). Three attempts were made with the arm unsupported and the elbow flexed at 90°,^39^ and the highest value was used for analysis. 

### Statistical analysis

Statistical analysis was performed with the Statistical Analysis System, version 9.4 (SAS Institute Inc., Cary, NC, USA), and GraphPad Prism, version 6.0 (GraphPad Software, Inc., San Diego, CA, USA). Depending on the data distribution, variables are expressed as frequency (percentage), mean (standard deviation), or median [interquartile range]. Data normality was assessed by the Shapiro-Wilk test. Correlations among the TUG tests (TUG_usual_ and TUG_fast_), number of daily steps, time spent in different postures (sitting, standing, and lying), and time spent in activities of different intensities (sedentary behavior, LPA, and MVPA) were made by using Spearman’s correlation coefficient. To establish cutoffs to identify physically inactive individuals, a ROC curve analysis was performed. The AUC, as well as the sensitivity and specificity of the proposed cutoffs, were calculated. Relevant characteristics for ILD patients were compared on the basis of the proposed cutoff points by using the unpaired t-test or the Mann-Whitney test, depending on the data distribution. Values of p < 0.05 were considered significant. 

## RESULTS

**Figure 1:**
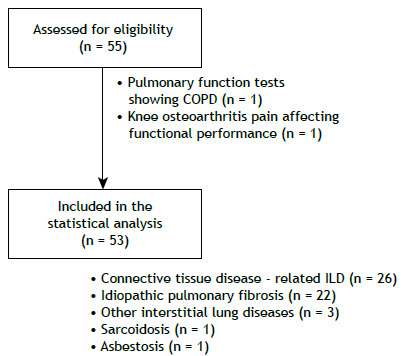
Study flowchart. ILD: Interstitial lung disease.

A total of 55 patients with ILD were assessed. Of those, 53 were included in the present study (Figure 1). Of the 53 ILD patients included in the study, 49% had connective tissue disease-associated ILD and 41% had idiopathic pulmonary fibrosis. The characteristics of the study participants and their performance on TUG_usual_ and TUG_fast_ are described in Table 1. Significant correlations were found among TUG_usual_ and TUG_fast_ (in s and % of predicted), number of steps/day, LPA, MVPA, and time spent standing (−0.59 < r < −0.31; p < 0.05 for all). A complete description of correlations is provided in Table 2. 

**Table 1:** Characteristics of the study sample.^a^

Variable	Patients with ILD (n = 53)
Sex, female	26 (49)
Age, years	60 ± 11
BMI (kg/m^2^)	27 [25-30]
*Lung function*	
FVC, % of predicted	68 ± 17
FEV_1_, % of predicted	69 ± 18
FEV_1_/FVC ratio	84 [78-87]
DL_CO_, % of predicted	45 ± 18
*Physical activity in daily life*	
Sedentary behavior, min/day	742 ± 178
LPA, min/day	298 ± 92
MVPA, min/day	8.8 [2.8-14.0]
Steps, n/day	4,874 ± 1,858
Time spent standing, min/day	290 ± 82
Time spent lying, min/day	295 [228-337]
Time spent sitting, min/day	441 ± 100
*Functional performance*	
TUG_usual_, s % of predicted	9.7 ± 1.4 102 [95-114]
TUG_fast_, s % of predicted	7.7 ± 1.1 104 [95-119]
Exercise capacity	
6MWD, m	449 ± 101
6MWD, % of predicted	81 ± 20
Peripheral muscle strength	
Quadriceps strength, N	271 [227-309]

ILD: interstitial lung disease; LPA: physical activity; MVPA: moderate to vigorous physical activity; TUG_usual_: timed “up and go” at a usual pace; TUG_fast_: timed “up and go” at a fast pace; and 6MWD: six-minute walk distance. ^a^Data presented as n (%), mean ± SD, or median [IQR].

**Table 2:** Correlation of the timed “up and go” test performed at a usual pace and at a fast pace with physical activity in daily life variables.

Variable	TUG_usual_		TUG_fast_	
	seconds	% of predicted	seconds	% of predicted
Sedentary behavior, min/day	0.02	0.01	0.12	0.16
LPA, min/day	−0.46*	−0.40*	−0.42*	−0.30*
MVPA, min/day	−0.59*	−0.55*	−0.47*	−0.35*
Steps, n/day	−0.58*	−0.45*	−0.51*	−0.31*
Time spent standing, min/day	−0.36*	−0.22	−0.25	−0.08
Time spent lying, min/day	0.12	0.06	0.05	0.04
Time spent sitting, min/day	−0.09	−0.13	−0.18	−0.29

TUG_usual_: timed “up and go” at a usual pace; TUG_fast_: timed “up and go” at a fast pace; LPA: light physical activity; and MVPA: moderate to vigorous physical activity. *p < 0.05.

**Figure 2:**
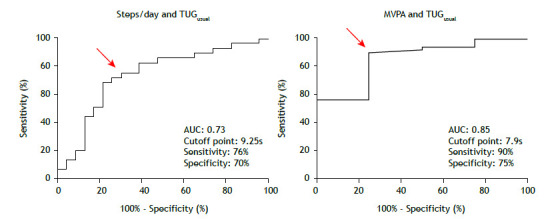
Area under the curve for the timed “up and go” test performed at a usual pace (TUG_usual_): cutoff points for the number of steps/day and time/day spent in moderate to vigorous physical activity (MVPA).

Twenty-nine ILD patients (55% of the sample) performed < 5,000 steps/day, and 49 (92%) performed < 30 min/day of MVPA. Analysis of the ROC curves showed that cutoffs of ≥ 9.25 s and ≥ 7.9 s on the TUG_usual_ test can identify physically inactive individuals on the basis of 5,000 steps/day (AUC: 0.73; sensitivity, 76%; specificity, 70%) and 30 min/day of MVPA (AUC: 0.85; sensitivity, 90%; specificity, 75%; Figure 2). Analysis of the ROC curves for TUG_fast_ in seconds and for TUG_usual_ and TUG_fast_ in % of predicted showed that they were less effective in distinguishing between physically active and physically inactive individuals (AUC: 0.60-0.79; sensitivity, 61-74%; specificity, 55-87%). Table 3 shows a complete description of sensitivity, specificity, and AUC values for TUG_usual_ and TUG_fast_ in seconds and % of predicted. 

**Table 3:** Cutoff points, area under the curve, sensitivity, and specificity for the timed “up and go” test performed at a usual pace and at a fast pace to identify physically inactive individuals with interstitial lung disease.

Variable	TUG_usual_		TUG_fast_	
	seconds	% of predicted	seconds	% of predicted
< 5,000 steps/day				
Cutoff point	9.25	101%	7.6	104%
AUC	0.73	0.65	0.69	0.60
95% CI	059-0.87	0.50-0.80	0.54-0.85	0.44-0.76
Sensitivity	76%	62%	66%	61%
Specificity	70%	64%	74%	55%
p	0.003	0.05	0.01	0.20
< 30 min/day of MVPA				
Cutoff point	7.9	90%	8.2	90%
AUC	0.85	0.79	0.69	0.79
95% CI	0.67-1.0	0.63-0.95	0.29-1.0	0.63-0.95
Sensitivity	90%	67%	74%	66%
Specificity	75%	87%	75%	87%
p	0.02	0.09	0.21	0.09

TUG_usual_: timed “up and go” at a usual pace; TUG_fast_: timed “up and go” at a fast pace; and MVPA: moderate to vigorous physical activity. *p < 0.05.

Thirty ILD patients (57% of the sample) showed a performance of ≥ 9.25 s on the TUG_usual_ test (the cutoff for < 5,000 steps/day), whereas 48 (91%) showed a performance of ≥ 7.9 s (the cutoff for < 30 min/day of MVPA). Table 4 shows the demographics of the sample, as well as data on exercise capacity, muscle strength, lung function, and PADL for the cutoff of ≥ 9.25 s. The ILD patients who performed worse on the TUG_usual_ test (i.e., ≥ 9.25 s) showed a lower number of steps/day (p < 0.001), less time spent in LPA (p < 0.001), less time spent in MVPA (p = 0.0008), and less time spent in a standing position (p = 0.0005), as well as more time spent in a lying position (p = 0.003), than did those who performed better. They also performed worse on the 6MWT and on the peripheral muscle strength assessment, with no significant differences in demographics and lung function. 

**Table 4:** Characteristics of the interstitial lung disease patients who performed worse (≥ 9.25 s) or better (< 9.25 s) on the timed “up and go” test performed at a usual pace, as assessed by ROC curves.^a^

Variable	Worse (slower) performance (n = 30)	Better (faster) performance (n = 23)	p
Sex, female	18 (60)	8 (35)	0.06
Age, years	64 [57-70]	56 [47-67]	0.14
BMI, kg/m^2^	27 [25-30]	27 [23-30]	0.58
*Lung function*			
FVC, % of predicted	66 ± 21	69 ± 12	0.58
FEV_1_, % of predicted	69 ± 21	69 ± 14	0.93
FEV_1_/FVC ratio	84 ± 6	89 ± 9	0.05
DL_CO_, % of predicted	42 ± 19	48 ± 18	0.29
*Physical activity in daily life*			
Sedentary behavior, min/day	756 ± 195	720 ± 159	0.42
LPA, min/day	254 ± 88	360 ± 78	< 0.001
MVPA, min/day	4.1 [1.1-9.2]	10.4 [8.8-21.7]	0.0008
Steps, n/day	4,041 ± 1,787	6,001 ±1,414	< 0.001
Time spent standing, min/day	255 ± 69	333 ± 78	0.0005
Time spent lying time, min/day	334 ± 137	266 ± 59	0.003
Time spent sitting time, min/day	428 ± 109	456 ± 87	0.33
*Exercise capacity*			
6MWD, m	389 ± 68	532 ± 79	< 0.001
6MWD, % of predicted	75 ± 14	90 ± 23	< 0.001
*Peripheral muscle strength*			
Quadriceps strength, N	271 [227-309]	417 [284-477]	0.001
Handgrip strength, kgf	21 [18-25]	28 [22-35]	0.005

LPA: physical activity; MVPA: moderate to vigorous physical activity; and 6MWD: six-minute walk distance. ^a^Data presented as n (%), mean ± SD, or median [IQR].

## DISCUSSION

In the present study, the TUG test was moderately correlated with PADL variables (i.e., number of steps/day, LPA, MVPA, and time spent standing) in patients with ILD. Although TUG_usual_ in seconds identified inactivity in ILD patients, TUG_fast_ did not. Additionally, our results show that a cutoff of ≥ 9.25 s can distinguish physically inactive individuals (i.e., those who walk < 5,000 steps/day) from individuals who are more active. Furthermore, we found that individuals who performed worse on the TUG_usual_ test (i.e., ≥ 9.25 s) were less active, more deconditioned, and weaker than those who performed better. 

To date, only two studies^8^ ^,^ ^22^ ^)^ investigated the performance of ILD patients on TUG_usual_. The mean duration of the test was 9.6 s in one of the studies and 9.8 s in the other, a finding that is consistent with those of the present study. The two aforementioned studies also investigated the correlations between performance on the TUG test and clinical outcomes.^8^ ^,^ ^22^. One of the studies^22^ found that TUG_usual_ correlated weakly with quadriceps femoris strength (r = −0.28; p = 0.164) and the 6MWD (r = 0.37; p = 0.062), whereas the other found that TUG_usual_ correlated moderately with quadriceps strength (r = −0.48; p < 0.05) and the 6MWD (r = −0.69; p < 0.05).^8^ The results of the present study expand the current knowledge regarding clinical associations of the TUG test, showing its association with PADL variables. In fact, the present study appears to be the first to show that a poor performance on the TUG test is associated with worse patterns of PADL in ILD patients. Given that PADL plays an important role in the morbidity and mortality of ILD,^4^ identification of a poor performance on the TUG test could guide clinicians in their decision to seek a more specific assessment of PADL in this population. 

The predictive power of the TUG test for different clinical outcomes has been demonstrated in patients with COPD.^25^ The aforementioned study^25^ showed that patient performance on the TUG test (i.e., > 11.2 s) has acceptable specificity and sensitivity to predict lower exercise capacity (i.e., a 6MWD of < 350 m). Despite the differences between the cutoffs for COPD and those for ILD proposed in the present study, there is currently no cutoff point to identify (in)activity in patients with ILD. Although functional performance tests such as the Glittre Activities of Daily Living test have been shown to correlate moderately with total energy expenditure,^18^ no other PADL variable has been shown to correlate with such tests. To the best of our knowledge, the present study is the first to propose cutoffs to identify ILD patients as inactive on the basis of their performance on a simple functional test. 

The TUG_usual_ cutoffs of ≥ 9.25 s (for 5,000 steps/day) and ≥ 7.9 s (for 30 min/day of MVPA) can effectively stratify ILD patients into active and inactive individuals, whereas TUG_fast_ is less effective in identifying inactivity in ILD patients. Similar to accelerometry, TUG_usual_ assesses the speed at which patients with ILD perform daily tasks. This similarity might explain the lack of significance for TUG_fast_ in the present study. When accelerometers are unavailable in clinical practice, TUG_usual_ can be used in order to identify inactive ILD patients and should be preferred over TUG_fast_. In a study of patients with various respiratory diseases, velocities below 1.07 m/s on the four-meter gait speed test were found to be able to identify inactive individuals.^16^ However, patients with ILD show worse prognosis than do those with other respiratory diseases,^1^ and the aforementioned cutoff point might therefore not be suitable for ILD patients. According to the authors, one of the limitations of the aforementioned study^16^ was that only individuals who did not need oxygen therapy were included. This is perhaps more important for patients with ILD than for those with other diseases, given that oxygen desaturation during exertion is very common. The present study therefore expands on the knowledge of the impact of poor performance on functional tests in patients with ILD.^16^ ^,^ ^18^

In the present study, a worse performance on TUG_usual_ did not seem to discriminate between different severities of ILD (i.e., lung function). There was no difference between individuals with better or worse performance on the TUG test regarding lung function. This is in disagreement with previous studies reporting that a worse performance on functional tests is associated with worse clinical status in patients with chronic respiratory diseases.^13^ ^,^ ^16^ However, the results of the present study show that performance on the TUG test can identify lower exercise capacity and peripheral muscle strength. Although the proposed cutoffs appear to be able to identify inactive individuals, these findings cannot be extrapolated to a better/worse overall health status. Further studies are needed to confirm whether a worse performance on TUG_usual_ is also associated with other important outcomes in patients with ILD, such as health-related quality of life. 

The results of the present study should be interpreted with potential limitations in mind. First, the sample size was relatively small. Although this limits the external validity of our findings, our sample size is similar to those in previous studies examining PADL and functional performance tests in patients with ILD.^16^ ^,^ ^18^ In addition, it should be borne in mind that ILD is less prevalent than other respiratory diseases such as COPD and asthma, making recruitment more difficult. Given that the sample size also has an impact on ROC curves, larger samples might be useful to strengthen our findings regarding the discriminative capacity of TUG_usual_ to identify physically inactive individuals. Although TUG_usual_ can estimate inactivity on the basis of the number of steps/day and the time/day spent in MVPA, it should not be the method of choice when other, more accurate methods are available to assess PADL. 

Second, TUG_usual_, TUG_fast_, and the 6MWT were performed within the same period, and the effort required to complete the TUG test may have influenced patient performance on the 6MWT. However, the order of execution was standardized, the protocol adhered to the recommended rest intervals, and baseline values of heart rate, SpO_2_, dyspnea, and sensation of fatigue were controlled before the two 6MWTs were performed. 

Third, the individuals included in the present study do not cover the entire spectrum of severities and subgroups of ILD. Most of the study participants had connective tissue disease-associated ILD or idiopathic pulmonary fibrosis (49% and 41%, respectively). Although the disease can influence progression, pulmonary manifestations, and extrapulmonary manifestations, the results for these two subgroups were similar in the present study. In addition, only one patient required oxygen therapy during the TUG tests, and only 8% of the study sample met the criterion of performing at least 30 min/day of MVPA. This may have influenced the findings regarding MVPA in the present study. Therefore, the cutoff points for TUG_usual_ might not accurately reflect PADL levels in ILD patients with different characteristics, in those with different disease severities, and in those receiving oxygen therapy. Future prospective studies might be able to address whether the cutoffs proposed in the present study are related to worsening lung function and are valid to assess negative clinical outcomes such as hospitalization and mortality. 

In conclusion, performance on TUG_usual_ and TUG_fast_ correlates moderately with PADL in patients with ILD. A TUG_usual_ performance ≥ 9.25 s appears to be able to identify physically inactive individuals in this population. 
